# The impact of COVID-19 on the indigenous peoples related to air and road networks and habitat loss

**DOI:** 10.1371/journal.pgph.0000166

**Published:** 2022-03-07

**Authors:** Luciana Cristina Vitorino, Ueric José Borges de Souza, Mateus Neri Oliveira Reis, Layara Alexandre Bessa

**Affiliations:** 1 Laboratory of Agricultural Microbiology, Goiano Federal Institute, Rio Verde—GO, Brasil; 2 Bioinformatics and Biotechnology Laboratory, Federal University of Tocantins, Gurupi—TO, Brasil; 3 Laboratory of Plant Mineral Nutrition and Center of Excellence in Exponential Agriculture—CEAGRE, Goiano Federal Institute, Rio Verde—GO, Brasil; University of Louisville School of Public Health and Information Sciences, UNITED STATES

## Abstract

The vegetation loss in the Brazil’s Legal Amazon (BLA) in 2020 corresponds to the highest loss observed in a decade, caused by the intensification of fires, mineral extraction activities, and other pressures. The possibility of earning from illegal activities such as deforestation and mining attracts the population to indigenous territories, while fires aggravate respiratory problems and enhance the current COVID-19 crisis. Furthermore, the BLA’s road network is usually related to increased deforestation and fires in its areas of influence, and airports are known to contribute to spreading COVID-19 infections worldwide. Therefore, we decided to evaluate the effect of characteristics of Special Indigenous Health Districts (DSEIs) (including population, number of airports, and extent of the road network) and vegetation loss rates (deforestation, and area of vegetation lost by fires and mining) on the number of COVID-19 cases and deaths among the indigenous population in DSEIs in the BLA. We observed a positive correlation between the number of cases and deaths and the number of Indigenous Primary Healthcare Units, suggesting that many of these units did not increase appropriate activities for prevention and protection from COVID-19 in the DSEIs. The DSEIs with larger air transport and road networks were more affected by COVID-19. These networks constituted critical mechanisms for facilitating the spread of SARS-CoV-2 in the BLA. Additionally, we noted that changes that impact the landscape of DSEIs, such as fires and mining, also impact legal indigenous areas (IAs). Thus, IAs are not spared from exploratory processes in the district’s landscape. Models that associate the air transport and road networks with the transformation of the landscape in IAs from burning or mining can explain the number of indigenous people who died due to COVID-19. These results are particularly important given the current disruptive scenario imposed by the Brazilian government on critical institutions that detect and fight fires in indigenous lands and the policies enacted to combat COVID-19 in Brazil, which are based on denying isolation measures and delaying vaccinations.

## Introduction

Indigenous health in Brazil is managed by 34 Special Indigenous Health Districts (DSEIs) coordinated by the Special Secretariat for Indigenous Healthcare (SESAI) of the Ministry of Health in the subsystem of the Unified Health System (SUS). These DSEIs comprise a territorial and demographic base under a clearly defined health responsibility, which is determined by sociocultural, geographic, and epidemiological criteria. For primary healthcare, DSEIs have their own service network in indigenous lands, and for moderately and highly complex medical procedures, they coordinate with the regional network [1]. The service structure of the DSEIs consists of indigenous primary healthcare units, base centers, and indigenous healthcare houses. The actions attributed to the DSEIs are significantly relevant, given the increasing number of health problems faced by indigenous people: high infant and maternal mortality; high incidence of infectious diseases; malnutrition and delayed growth; reduced life expectancy at birth; smoking-related illnesses and deaths; social problems, alcohol and drug-related illnesses and deaths; accidents, poisonings, interpersonal violence, homicide, and suicide; obesity, diabetes, hypertension, cardiovascular disease, and chronic kidney disease (lifestyle diseases); diseases caused by environmental contamination (heavy metals, industrial gases, effluent residues); and infectious diseases caused by fecal contamination [2].

The importance of the activities in the DSEIs in indigenous healthcare and awareness becomes even more emblematic given the current scenario of the COVID-19 pandemic in Brazil, which has greatly concerned indigenous people, SESAI, and research institutions with the spatialization of infections and the impact of the spread of COVID-19 in Brazil’s Legal Amazon (BLA) [3,4]. In addition to COVID-19 in 2020, Brazilian indigenous territories were deeply affected by deforestation and fires. Brazil’s Legal Amazon Deforestation Monitoring Program (PRODES) estimated deforestation for 2020 at 11,088 km^2^ was based on 45% of the monitored area. This represents an increase of 47% and 9.5% compared to 2018 and 2019, respectively, and it is the highest index of the decade [5,6]. Mataveli et al. [7] warned of the emergence of a new deforestation hotspot in the Amazon rainforest located along the highway BR-319. Public bid notices to pave this highway were recently announced, and in the period after publication of the notices, from July to September 2020, warnings of deforestation and fire increased significantly in the highway’s area of direct influence.

In fact, the rise in deforestation in the Amazon rainforest not only compromises greenhouse gas reduction targets, but is also associated with a drier climate, which intensifies fire events [8]. Fires promote the emission of large amounts of smoke, which can affect the respiratory health of the population and aggravate the vulnerability of indigenous, traditional, and rural people to acute respiratory infections that are already among the most relevant causes of morbidity and mortality in indigenous populations, especially in infants [9–11]. In this scenario, the exposure of indigenous people to severe acute respiratory syndrome coronavirus 2 (SARS-CoV-2) increases with the growing threats to and invasions of their territories. Studies suggest that a probable relationship exists between air pollutants linked to fire, such as particulate matter, and COVID-19 infections, indicating that the large number of fires that occurred in BLA contributed to the aggravation of the current COVID-19 crisis [12,13].

The effect of airports in the spread of COVID-19 infections is also recognized. As COVID-19 spreads at different rates in different locations, Coelho et al. [14] found that population size and global connections, represented by the importance of countries in the global air transport network, were the main explanations for the initial growth rate of COVID-19 in different countries. On the other hand, accessibility to indigenous areas can increase illegal economic activities (presence of gold miners, loggers and land grabbers), increasing the risk of spreading the coronavirus [15]. Therefore, we decided to evaluate the hypothesis that population connections, given by the air and road networks, contributed to the dissemination of COVID-19 cases in DSEIs in BLA in 2020. In this region, the relationship between the existence of roads and the increase in deforestation and fires [16,17] in the areas of influence of these roads is classically reported. Our other hypothesis is that DSEIs subjected to different levels of vegetation loss were differentially affected by the COVID-19 pandemic in 2020, before initiation of vaccination.

## Materials and methods

### Data on indigenous health and DSEIs

**Fig 1:**
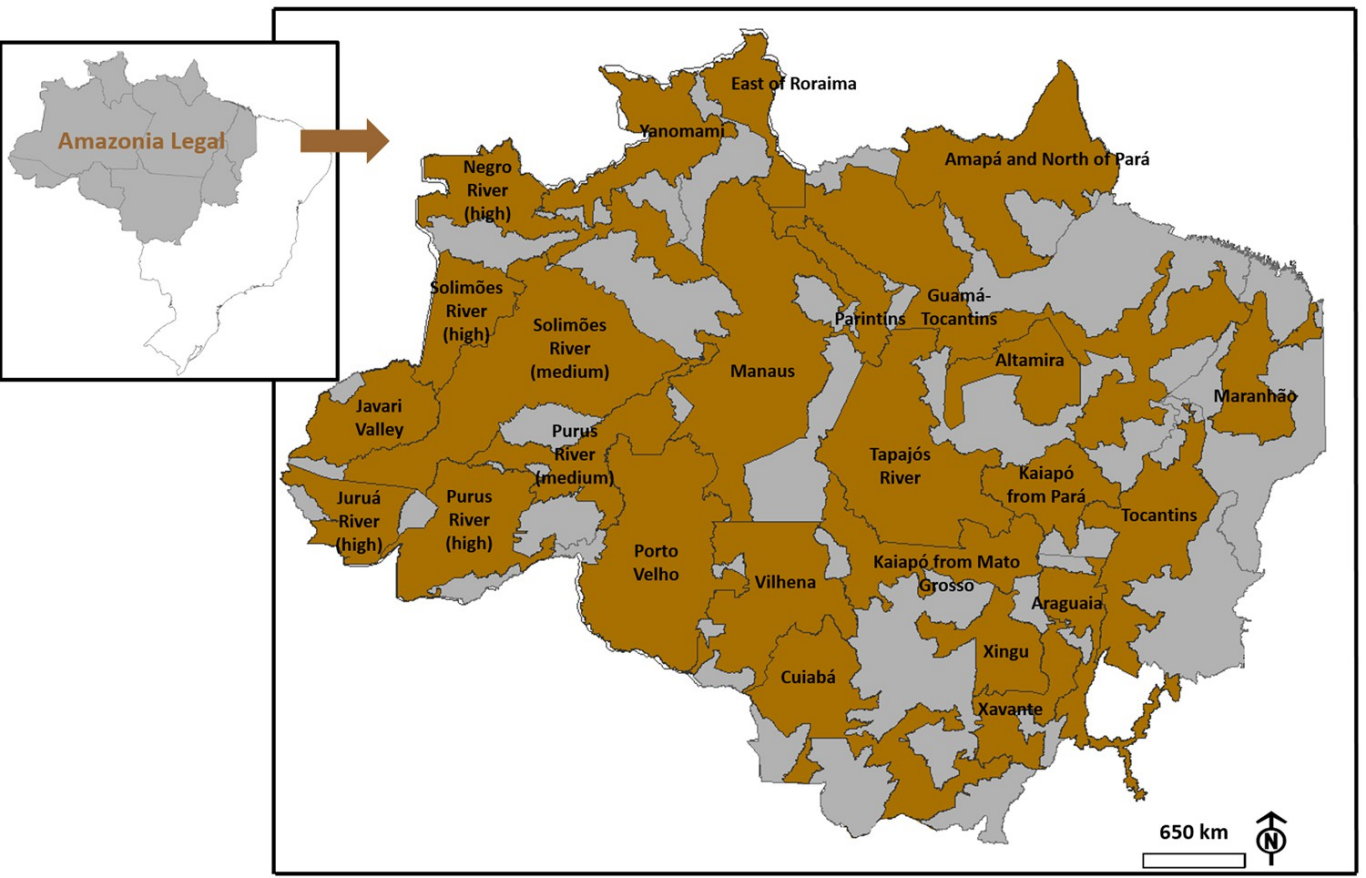
Geographical distribution of the 25 Special Indigenous Health Districts (DSEIs) in Brazil’s Legal Amazon.

The data on the number of COVID-19 cases and deaths among indigenous people in 25 DSEIs in BLA (Fig 1) were collected from a weekly report (*Situação do COVID-19 na Amazônia Legal*; COVID-19 Situation in BLA) issued by the Operations and Management Center of the Amazonian Protection System (CENSIPAM), which is available at https://www.gov.br/defesa/pt-br/assuntos/censipam/publicacoes/mapas-covid-19. The data, last updated at 8 am on January 13, 2021, was used because the numbers were under the influence of 2020 (natural vegetation loss was analyzed for the same year) and data obtained in the bulletins issued from this date would already be under the influence of the vaccination process, which initiated on January 17^th^ in Brazilian cities and on January 19^th^ in indigenous tribes. We assume the data made available by CENSIPAM as real data and choose not to consider under-reporting due to the difficulty of obtaining accurate data on these rates and because we believe they are highly variable among DSEIs. Alternatively, data on the total population, number of ethnicities, number of villages, number of indigenous primary healthcare units (UBSIs), and area (km^2^) were obtained from the indigenous healthcare site of the Ministry of Health (Brazil) (https://saudeindigena1.websiteseguro.com/coronavirus/dsei/).

For each DSEI, with geographic distribution obtained through the layer of the DSEIs of Brazil (https://www.gov.br/funai/pt-br/atuacao/terras-indigenas/geoprocessamento-e-mapas), we verified the total number of airports, total road area, and the number of cities with 10,000, 20,000, 50,000, 100,000, 200,00, and 300,000 inhabitants. The list of municipalities in BLA was retrieved from a georeferencing database (*.shp) available in the data system of the Brazilian Institute of Geography and Statistics (IBGE) at: https://www.ibge.gov.br/geociencias/cartas-e-mapas/mapas-regionais/15819-amazonia-legal.html?=&t=downloads. The population estimates by municipality for the year 2020 were collected from IBGE’s data system at: https://www.ibge.gov.br/estatisticas/sociais/populacao/9103-estimativas-de-population.html?=&t=downloads. This information was grouped in the layers for the area occupied by DSEIs using the ArcMap Union tool in the ArcGis 10.5 software [18]. The number of cities by population was quantified using the ArcMap Summarize tool. Subsequently, these cities were organized based on population: over 10,000, 20,000, 50,000, 100,000, 200,000, and 300,000 (for more details on the dataset, see the S1 Data).

The air transport network in the DSEIs was obtained by the sum of the public and private international and national airports sampled within the area of each DSEI. These airport samples were collected from a georeferencing database (*.shp), which is available at https://antigo.infraestrutura.gov.br/component/content/article/63-bit/5124-bitpublic.html#mapaero. The total road network (TRN, km) in the DSEIs was calculated by adding the extent of the federal, federally granted, and state road networks found within the DSEIs from the georeferencing database for these highways, which is available at https://antigo.infraestrutura.gov.br/component/content/article/63-bit/5124-bitpublic.html#maprodo. Therefore, the layers corresponding to these networks were superimposed on the DSEI area layer using the ArcMap Intersect (Analysis) tool in the ArcGis software. The extent of the network was calculated for each district separately using the Summarize tool.

### Vegetation loss

The data on deforested areas and deforested legal indigenous areas (IAs) within the DSEI (km^2^) were collected from a georeferencing database provided by the PRODES project (Ministry of the Environment–MMA and Brazilian Institute of the Environment and Renewable Natural Resources–IBAMA) with the information on the annual deforested area in 2020 (http://terrabrasilis.dpi.inpe.br). This information was plotted within the areas currently occupied by DSEIs and areas identified as IAs in the BLA using the Intersect tool (Analysis). To calculate the sum of the polygons corresponding to the deforested areas for each DSEI and for the IAs within each DSEI, we used the ArcMap Summarize tool.

Moreover, the data on areas deforested due to burning and mineral extraction activity (km^2^) within the DSEIs and IAs in the BLA (fires-DSEI and fires-IA; mining-DSEI and mining-IA) were collected from a georeferencing database published for BLA at http://terrabrasilis.dpi.inpe.br/app/map/alerts?hl=pt-br. The data on burn scars and mining for 2020 were retrieved from this database. These data were plotted within the DSEI area and the areas identified as IAs in BLA using the Intersect tool (Analysis). The Summarize tool was utilized to summarize the data, which allowed us to determine the sum of the polygons corresponding to the areas deforested by fires and mineral extraction activities. The results were obtained for each DSEI and for the IAs within each DSEI.

### Statistical analysis

Initially, all variables were treated independently and evaluated using Pearson’s correlation coefficient. The strength of the relationship was analyzed by the values for r and by the significance of the interaction at 5% and 1% probability. This analysis was conducted using the “Corrplot” package in R, version 4.0.4 [19,20].

We employed Generalized Linear Mixed Models (GLMMs) to examine the effects of the changes in the landscape and characteristics of the DSEIs and the number of airports and TRN on the number of COVID-19 cases and deaths among the indigenous population in BLA in 2020. Before constructing the models, we tested the multicollinearity between the variables with the “Car” package [21] implemented in R, version 4.0.4, and removed variables with vif >5 from the models. The deforested area in DSEIs and in IAs, as well as the area of vegetation lost due to fire and mining activity in IAs were used as the explanatory variables. We analyzed the number of airports and TRN separately in the models, which were included as random factors, and the other explanatory variables were fixed factors.

We also developed models to determine whether random effects can also account for the response variables. These models considered only the random effects on the number of cases and deaths, discarding the effects of the size of the deforested area in DSEIs and IAs, and of the size of the area of vegetation loss due to fire and mining activity in IAs. We utilized the MCMCglmm package [22] to perform the analyses in a Bayesian framework with the Markov Chain Monte Carlo algorithm. A total of 80,000 iterations with 20,000 burn-in chains and a Gaussian distribution were used. The Akaike information criteria (AIC) were used to select the best model: the model with the smallest AICc (the AIC corrected for sample size and the number of parameters), which was considered to be the most plausible for the explanation of the observed patterns [23]. The Delta AICci (ΔAICci, where i represents each model) was calculated as the difference between the AICc for the i^th^ model and the smallest AICc observed. We also determined Akaike’s weight (wAICc), which represents the relative contribution of the i^th^ model to the explanation of the observed pattern, given a set of competing models. The models with ΔAICc<2 were all considered equally plausible as explanations of the observed pattern [24].

## Results

**Fig 2:**
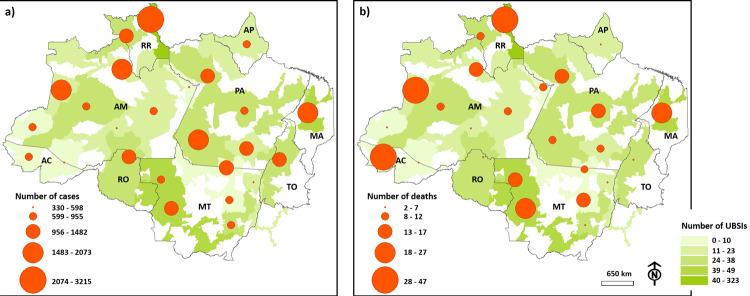
The number of COVID-19 cases (a) and deaths (b) of indigenous people in different Special Indigenous Health Districts (DSEIs) in Brazil’s Legal Amazon (BLA), depending on the number of indigenous primary healthcare units (UBSIs). The letters indicate the states in which the districts are located: RR = Roraima, AP = Amapá, MA = Maranhão, PA = Pará, AM = Amazonas, AC = Acre, RO = Rondônia, MT = Mato Grosso, and TO = Tocantins. Base layer obtained from: https://www.gov.br/funai/pt-br/atuacao/terras-indigenas/geoprocessamento-e-mapas.

In 2020, CENSIPAM reported a total of 27,034 cases and 354 deaths of indigenous people in the DSEIs in the BLA by COVID-19, with the highest number of cases observed in the Leste de Roraima DSEI (3215), followed by the Alto Rio Negro (2073) and Alto Rio Solimões (2001) DSEIs. The highest number of deaths was observed in the Leste de Roraima DSEI (47), followed by the Alto Rio Juruá (46) and Alto Rio Solimões (36) DSEIs. These DSEIs have 323, 0, and 14 UBSIs, respectively. The smallest number of cases was found in the Araguaia (330), Parintins (455), and Médio Rio Purus (496) DSEIs. The lowest number of deaths was reported in the Vale do Javari (2), Porto Velho (2), and Médio Rio Purus (5) DSEIs, which have 8, 26, and 13 UBSIs, respectively (Fig 2). The data demonstrate a true correspondence between the number of cases and the number of deaths. The DSEIs with a high number of cases tended to demonstrate a high number of deaths, and vice-versa.

**Fig 3:**
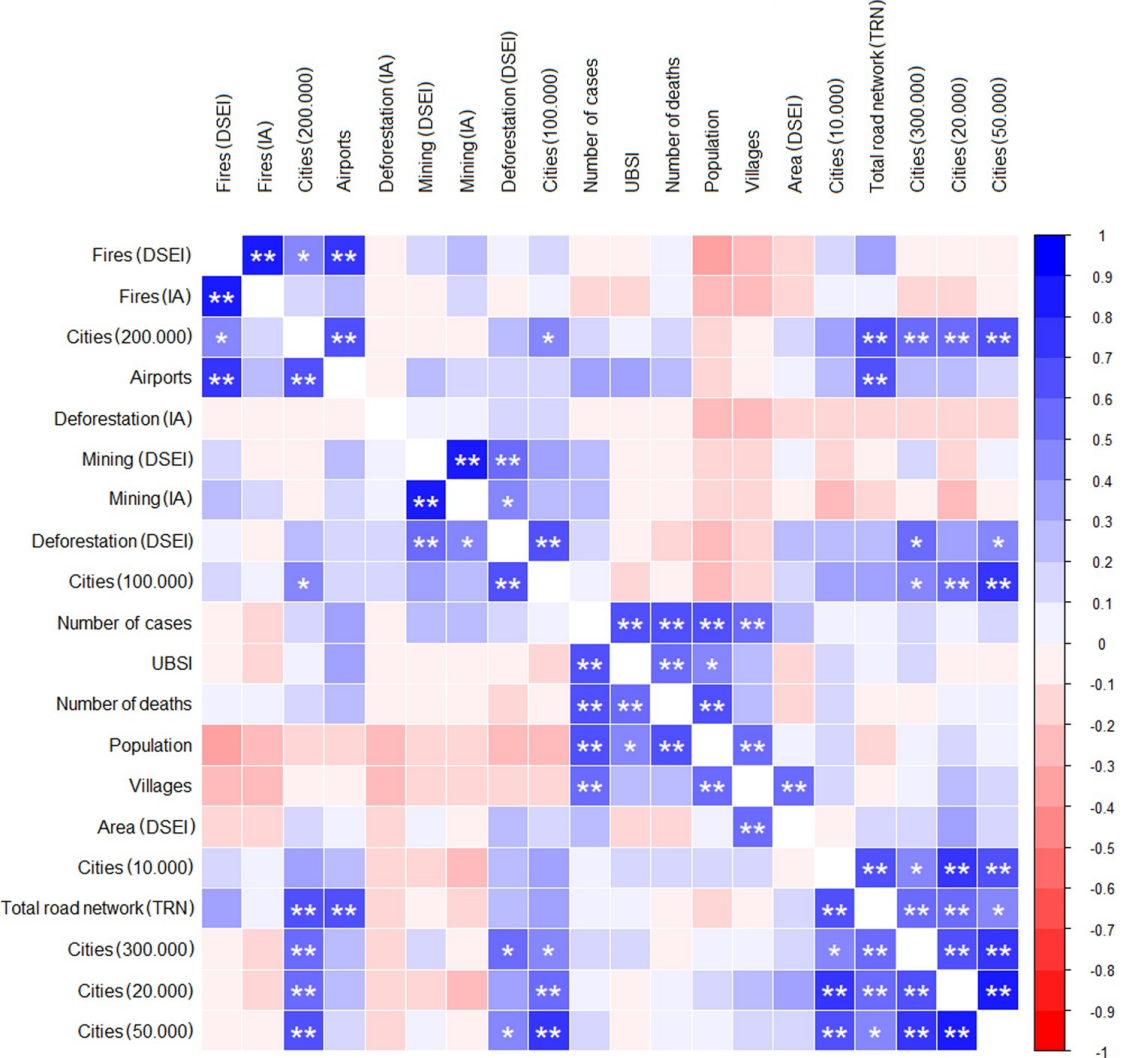
Correlation between indigenous health data (number of COVID-19 cases and deaths, number of UBSIs) and DSEIs (population; number of villages; area; number of cities with more than 10,000, 20,000, 50,000, 100,000, 200,000, and 300,000 inhabitants; total road network–TRN; and number of airports) and the landscape modification data (deforested area in DSEIs and in indigenous areas (IAs) within the DSEIs, deforested area due to fire and mining activity in DSEIs and IAs) observed for Brazil’s Legal Amazon in 2020. * significant at 5% probability; ** significant at 1% probability.

The number of COVID-19 cases and deaths in the DSEIs were found to have a positive correlation with the indigenous population density in the DSEIs. However, no correlation was observed between these variables and any other characteristics of the DSEIs or landscape variables analyzed. The analysis of the interaction between other variables showed that the size of the area of vegetation lost to fires had a positive correlation with the size of the area of vegetation lost to fires within IAs, the number of cities with over 200,00 inhabitants, and the number of airports found in the DSEI (Fig 3).

The size of the deforested area in DSEIs was found to be positively correlated with the area corresponding to vegetation lost due to mining activities in DSEIs and in IAs and the number of cities with 50,000, 100,000, and 300,000 inhabitants. However, vegetation losses due to mining activity in DSEIs also incur losses within IAs.

The DSEIs with the largest number of cities (with more than 10,000, 20,000, 50,000, 200,000, and 300,000 inhabitants) concentrate the largest TRN. In addition to a large road network, DSEIs that concentrate cities with more than 200,000 inhabitants also concentrate many airports. However, a high positive correlation was found between the accessibility measures; thus, DSEIs with a high TRN also have many airports.

The models consisting of only the number of airports or the extent of the TRN provide a plausible explanation for the number of COVID-19 cases observed in the DSEIs. However, when these variables are associated with mining-IA, it can also explain the incidence of this severe acute respiratory syndrome in the indigenous population in DSEIs. These models, nevertheless, were not more explanatory than the null model.

When we analyzed the number of deaths, we noted that geographic factors (such as the number of airports and TRN) may individually explain the values, but some of the changes in the landscape (deforestation-DSEI, mining-IA, and fires-IA) also explain the values (Table 1).

**Table 1:** The models used to test the effect of the number of airports, total road network (TRN), deforested area within the DSEI, deforested area within indigenous areas (IAs) of the DSEI, natural vegetation area converted into mining area within IA, and natural vegetation area converted into a burned area within IA on the number of COVID-19 cases and deaths among indigenous people in the DSEIs of the Brazil’s Legal Amazon (BLA).

**Number of cases**					
**Model**	**ΔAICc**	**wAIC**	**K**	**AIC**	**Pr (>F)**
**Null model**	**0.0**	**0.407**	**2**	**397.00**	**-**
**Cases vs. Airports**	**1.4**	**0.198**	**3**	**398.44**	**0.47900**
**Cases vs. Airports + Mining (IA)**	**1.9**	**0.147**	**4**	**399.03**	**0.42190**
Cases vs. Airports + Deforestation (DSEI)	3.1	0.086	4	400.10	0.67380
Cases vs. Airports + Fires (IA)	3.2	0.083	4	400.18	0.69820
Cases vs. Airports + Deforestation (IA)	3.3	0.079	4	400.27	0.72710
**Null model**	**0.0**	**0.420**	**2**	**395.00**	**-**
**Cases vs. TRN**	**1.5**	**0.200**	**3**	**397.00**	**0.50270**
**Cases vs. TRN + Mining (IA)**	**1.9**	**0.147**	**4**	**400.00**	**0.55670**
Cases vs. TRN + Deforestation (DSEI)	3.0	0.092	4	400.12	0.68980
Cases vs. TRN + Fires (IA)	3.4	0.081	4	400.20	0.69700
Cases vs. TRN + Deforestation (IA)	3.5	0.075	4	400.30	0.71340
**Number of deaths**					
**Model**	**ΔAICc**	**wAIC**	**K**	**AIC**	**Pr (>F)**
**Deaths vs. Airports**	**0.0**	**0.330**	**3**	**197.89**	**0.05728**
**Deaths vs. Airports + Deforestation (DSEI)**	**1.3**	**0.170**	**4**	**199.16**	**0.12370**
**Deaths vs. Airports + Mining (IA)**	**1.8**	**0.130**	**4**	**199.66**	**0.15460**
**Deaths vs. Airports + Fires (IA)**	**1.9**	**0.125**	**4**	**199.83**	**0.16650**
Deaths vs. Airports + Deforestation (AI)	2.0	0.123	4	199.87	0.16960
Null model	3.0	0.120	2	199.91	-
**Deaths vs. TRN**	**0.0**	**0.300**	**3**	**197.90**	**0.05478**
**Deaths vs. TRN + Deforestation (DSEI)**	**1.4**	**0.180**	**4**	**199.20**	**0.13280**
**Deaths vs. TRN + Mining (IA)**	**1.9**	**0.130**	**4**	**199.70**	**0.16490**
Deaths vs. TRN + Fires (IA)	2.0	0.124	4	199.80	0.16950
Deaths vs. TRN + Deforestation (AI)	2.0	0.123	4	199.89	0.16990
Null model	3.0	0.120	2	199.92	-

The models with **Δ** AICc < 2.0 are in bold type. AIC = Akaike value; AICc = AIC corrected by sample size and number of parameters in the model; wAIC = Akaike weight; K = number of parameters. *Pr* is the probability of the model in relation to the null model, obtained by the F-test.

**Fig 4:**
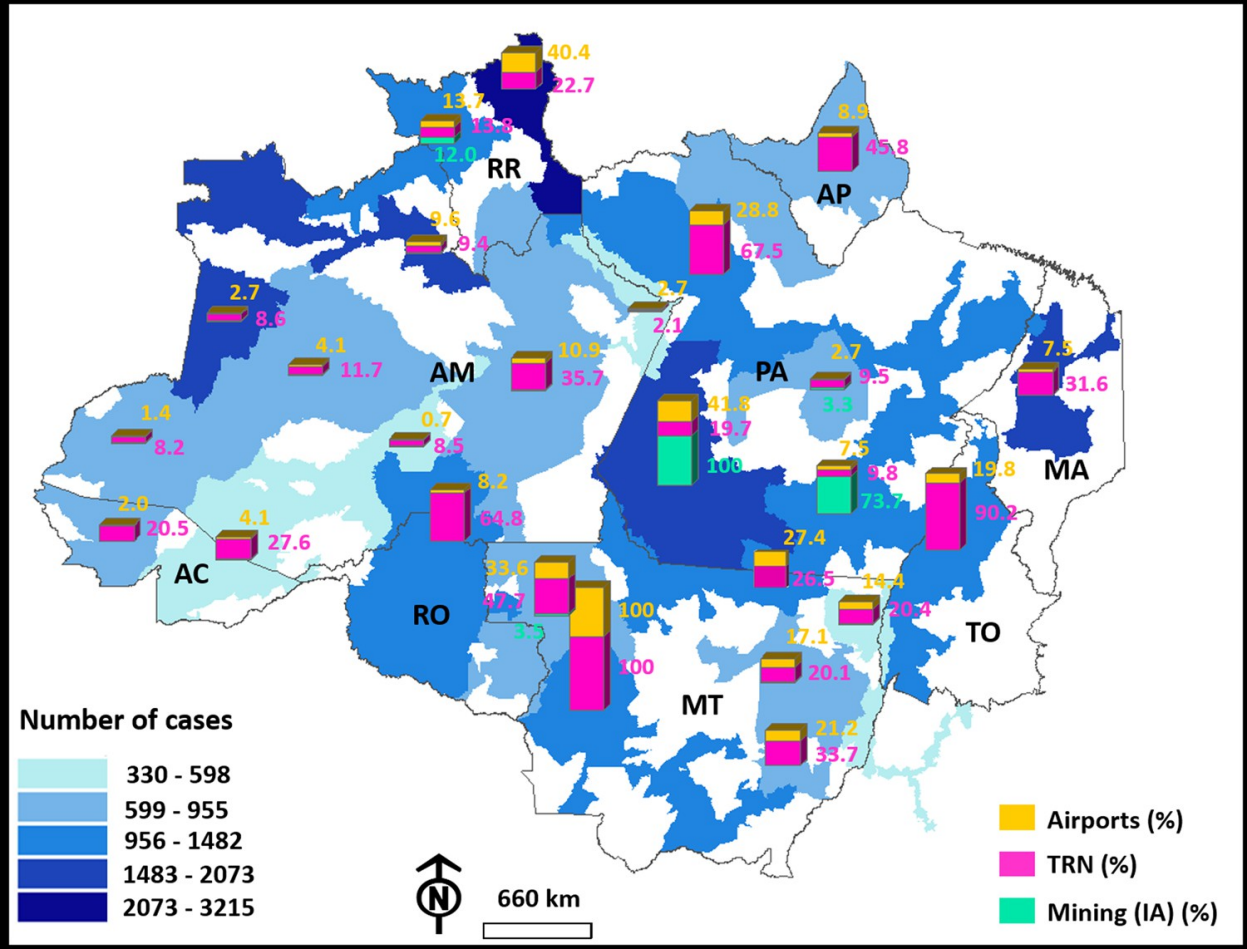
The number of COVID-19 cases among indigenous people in the DSEIs of Brazil’s Legal Amazon as a function of the relative percentage of air transport network, total road network (TRN), and natural vegetation area converted into mining areas within the IAs of the DSEIs. Base layer obtained from: https://www.gov.br/funai/pt-br/atuacao/terras-indigenas/geoprocessamento-e-mapas.

**Fig 5:**
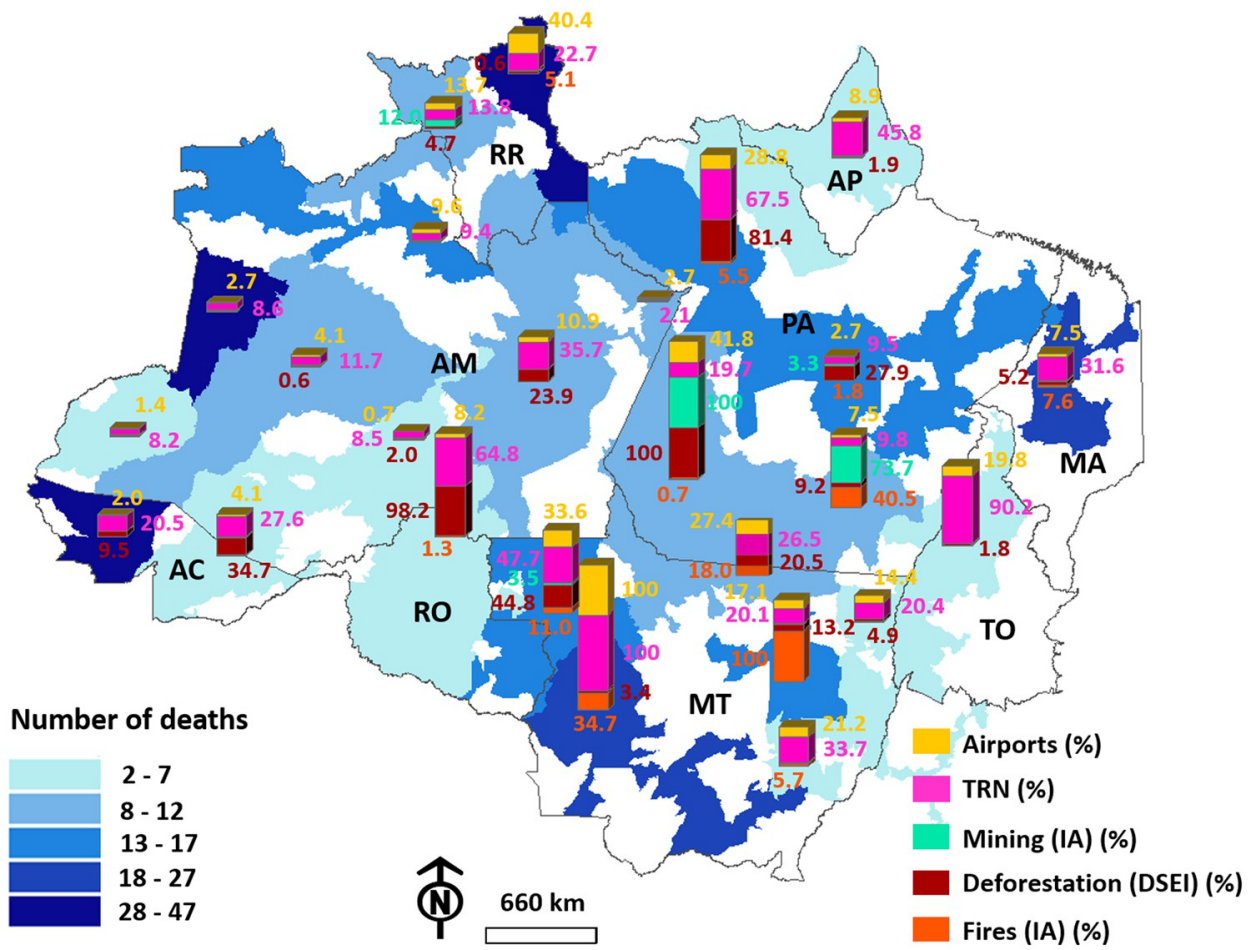
The number of COVID-19 deaths among indigenous people in the DSEIs of Brazil’s Legal Amazon (BLA) as a function of the relative percentage for air transport network, total road network (TRN), deforested area within the DSEI, natural vegetation area converted into mining area within IAs, and natural vegetation area converted into a burned area within IAs of the DSEI. **Base layer obtained from: https://www.gov.br/funai/pt-br/atuacao/terras-indigenas/geoprocessamento-e-mapas**.

Based on the results obtained from the mixed models, the relationship between the number of cases and the number of airports, TRN, and mining-IA was plotted. It became evident that the DSEIs with the highest numbers of airports, such as Cuiabá, Rio Tapajós, and Leste de Roraima, also presented a high number of COVID-19 cases and deaths among the indigenous population. There were 1297 cases and 24 deaths in Cuiabá, 1991 cases and 12 deaths in Rio Tapajós, and 3215 cases and 47 deaths in Leste de Roraima (Figs 4 and 5). Conversely, the DSEIs with low number of airports, such as Médio Rio Purus (1), Parintins (4), and Alto Rio Purus (6), demonstrated a low rate of COVID-19 cases and deaths among indigenous populations in 2020. There were 496 cases and 5 deaths in Médio Rio Purus, 455 cases and 11 deaths in Parintins, and 598 cases and 5 deaths in Alto Rio Purus (Fig 4).

We detected a similar interaction between the number of cases and deaths and the extent of the TRN. We observed that DSEIs with little access by road networks, such as Parintins (±200 km long), Médio Rio Purus (±800 km), and Vale do Javari (±780 km), had a reduced number of cases and deaths (455, 496, and 772 cases and 11, 5, and 2 deaths, respectively). DSEIs with an extensive road network, such as Cuiabá and Guamá-Tocantins (±9500 and ±6400 km, respectively), tended to exhibit a high number of cases (1297 and 1482 cases, respectively) and deaths (24 and 17 deaths, respectively).

The number of indigenous people affected by COVID-19 was also concentrated in areas that had the highest percentages of natural vegetation converted into mining areas. The neighboring DSEIs, Rio Tapajós and Kaiapó do Pará, were the most affected by mining (±12 and ±9 km^2^, respectively) and by a high incidence of COVID-19 cases and deaths (1961 and 1204 cases). Nevertheless, in the sampled period, the DSEIs that presented the lowest incidences of COVID-19, such as Araguaia, Parintins, Médio Rio Purus and Alto Rio Purus (308, 375, 459 and 581, respectively), had zero forest conversion rates in the mining area.

Based on the mixed models, we also plotted the number of COVID-19 deaths of indigenous people against the deforestation rate in the DSEIs. The DSEIs significantly affected by deforestation, such as Vilhena and Guamá-Tocantins (±600 and ±1060 km^2^, respectively), tended to present a high number of COVID-19 deaths (15 and 17, respectively) (Fig 5). Despite that, the DSEIs that had a low number of deaths from COVID-19, such as Vale do Javari, Amapá and Norte do Pará and Médio Rio Purus (2, 5 and 3, respectively), had low rates of habitat loss (±0.29, ±24.49. and ±1.23 km^2^) (Fig 5).

Following the observed pattern for deforestation, DSEIs, such as Cuiabá and Xingu, which had a high percentage of burned natural vegetation (±620 and ±1780 km^2^ of burned area, respectively) were observed to have a higher number of deaths (24 and 15 deaths, respectively). In contrast, in the DSEIs with the lowest number of deaths from COVID-19, such as Vale do Javari, Amapá and Norte do Pará, Médio Rio Purus, Alto Rio Purus and Araguaia (respectively, 2, 5, 3, 5 and 6), loss of vegetation with fires was nil.

## Discussion

The Leste de Roraima and Alto Rio Juruá were the DSEIs with the highest number of COVID-19 deaths of indigenous people in 2020. However, these DSEIs are the ones with the highest and lowest number of UBSIs, respectively. The positive correlation between the number of cases and deaths and the number of UBSIs suggests that the concentration of UBSIs did not increase appropriate activities for COVID-19 prevention and protection in the DSEIs. In Brazil, these units serve to take care of the integral health of the indigenous population and provide free access to medical care and prevention practices to socially vulnerable populations. The creation of SESAI in 2010 ensured a huge investment in hiring health professionals to work in the IAs, and then, as of 2013, the *Mais Médicos do Brasil* (More Doctors) Program brought 339 doctors into the IAs [25,26], which has always been a healthcare challenge in these territories. Therefore, the Indigenous Healthcare Subsystem is considered a victory of the indigenous, indigenist, and health movement in Brazil. With its expansion, it soon reached previously unattended regions and improved access to healthcare services, and it had a positive impact on health indicators in some regions. These units are essential in the implementation of preventive actions against child malnutrition, breast cancer, cytopathological diseases, or even suicide [27–31]. These units have demonstrated their importance in the dissemination and implementation of vaccination campaigns [32]. However, in recent years, the initial conception of the subsystem and the paradigm for special care have been deconstructed [33]. The Xingu Project team’s field reports indicate a change in the downward trend in infant mortality at different times [34]. The destabilization of this Subsystem became evident during this pandemic. Regarding the dissemination of the instructions adopted by the WHO, such as social distancing and the use of face masks to combat COVID-19 globally, the UBSIs appear to have not had relevance in IAs. It is also worth mentioning the case that occurred in the DSEI Alto Rio Solimões (third in the number of cases and deaths by COVID-19), in which the contact of local communities with an infected health professional [35] affected the initial rates of contagion and exposed the unpreparedness of this Subsystem in dealing with the possibility of iatrogenic transmission and containing the spread of the disease in indigenous lands.

The results of this study indicate that changes that impact the landscape of the DSEIs, such as fires and mining, also impact the legal IAs. Therefore, the IAs are not spared from exploratory processes in the district’s landscape. These data corroborate the conclusions made by BenYishay et al. [36] that the formalization of indigenous communities’ rights to land does not affect deforestation, as no improvements were verified, even in communities that receive support for surveillance and inspection. Thus, it is questionable whether indigenous land rights programs are justified in protecting against deforestation [37]. In Brazil, the government formalized the territorial rights of indigenous populations in its 1988 Constitution. Since then, indigenous lands have been formalized in more than one-fifth of the Brazilian Amazon. However, these results might be attributable to the locations of these lands, which are often located near the frontiers of deforestation expansion. Nevertheless, in these high-pressure areas, demarcation of indigenous lands can slow the process of vegetation loss [38]. Jusys [39] has shown that although indigenous lands were effective in preventing the highest percentage of deforestation during 2001–2004 and 2005–2008, deforestation prevention patterns began to change during 2009–2014, when strictly protected areas exceeded indigenous lands in terms of percentage of preserved forests. The critical component for deforestation in indigenous areas of the BLA may be found in the illegal leasing of these areas by farmers and ranchers who transform the landscape, promoting the loss of natural habitat and compromising soil quality. This practice has become common, as the current Brazilian government’s efforts to legalize mining and leasing activities in indigenous territories [40].

In general, deforestation practices in the Brazilian Amazon are related to the use of fire to remove natural vegetation and plant agricultural crops or pastures [41]. In recent years (particularly 2018, 2019, and 2020), annual deforestation rates in the Amazon have increased significantly, especially in protected areas such as IAs [42]. This increase is driven by the political turmoil between the opposing interests of rural producers and environmentalists [43] and the economic recession in Brazil [44]. The economic recession has favored deforestation and the expansion of agricultural activities in these areas, leading to increased stress on rights to indigenous lands and jeopardizing indigenous populations and other protected groups and traditional lifestyles in the Amazon [43]. If cases of deforestation and fires in the Amazon are related, the latter has contributed to elevating the risks of respiratory health and the air quality crisis in Brazil [11].

Our model, which included fires in IAs, was able to explain the number of COVID-19 deaths in the indigenous population in 2020. In a technical note, the Oswaldo Cruz Foundation [45] issued a warning about the impact that a large occurrence of fires in the Amazon (which reached record highs in 2020) combined with low humidity would have on the COVID-19 pandemic. According to the foundation, the particulate matter and toxic gases generated by burning biomass could reach cities, as well as riverside populations, quilombos, and indigenous lands hundreds of kilometers from the fires. Particulate matter has considerable inflammatory potential, which can aggravate inflammation associated with COVID-19 cases, and it constitutes a gateway for respiratory infections. This foundation proposed two priority areas for reinforcing the healthcare and fire control systems in the BLA: the Western Amazon, which does not have adequate road networks and healthcare infrastructure, and the arc of deforestation, mainly in northern Mato Grosso and southeastern Pará, with a higher number of vulnerable indigenous populations and incidence rates of COVID-19.

An unprecedented survey conducted by Global Forest Watch (https://www.globalforestwatch.org/map/) showed that Brazilian indigenous lands had been devastated by more than 115,000 fire outbreaks by October 2020. Satellite data also revealed that the indigenous lands of the Xingu (MT) were affected the most this year. Our data demonstrated that the incidence of these fires might be related to the number of COVID-19 deaths in this region. This scenario is relevant, given the disruption caused by the current Brazilian government to the crucial institutions that prevent fires in indigenous lands, such as Funai (National Indian Foundation) [46], and combat COVID-19, such as the Ministry of Health (Brazil).

We found that the DSEIs with significant air transport and road networks were more affected by COVID-19. Airports are known as excellent venues for the import and transmission of COVID-19 infections [47]. In Brazilian airports, no actions were taken to control and restrict the movement of individuals who had not been tested for SARS-CoV-2 for domestic transit, even when the community transmission of new variants had not yet been confirmed. The air transport and road networks have become real mechanisms that facilitate the spread of COVID-19 in the BLA. In general, the DSEIs with limited accessibility, such as Parintins and Médio Rio Purus, had few COVID-19 cases and deaths in the indigenous population. In a report on indigenous populations’ geographic and sociodemographic vulnerability to COVID-19 in Brazil, the Oswaldo Cruz Foundation [4] indicated that about 21.1% of the rural indigenous population in the BLA lived in municipalities with a high risk (>50%) for COVID-19. In fact, the internalization of the pandemic, which occurred more rapidly in areas having greater access by air or road, led to a significant increase in the number of indigenous people at high risk.

We observed that the development of larger cities with more than 100,000 inhabitants seems to encourage investments in road networks but also increases the environmental vulnerability of the DSEIs, exposing them to vegetation loss due to deforestation. Studies demonstrate that planned investments in highway paving and large infrastructure projects in the Brazilian Amazon accelerate forest loss [48]. Furthermore, the network increases access to the region for the purpose of logging or mining. According to Jusys et al. [49], official and unofficial roads are directly responsible for driving deforestation into remote areas of the BLA. Although low-volume roads facilitate transport to rural communities, logging and mining operations, and resource management [50], they accelerate deforestation, forest fragmentation, and degradation [51–53]. The same is the case with the spread of COVID-19 among indigenous people in the BLA.

We support the hypothesis that population connections established by the air and road network contributed to the spread of COVID-19cases in DSEIs in the BLA in 2020. Associated with this, the loss of vegetation related to the increase in fires and activity illegal mining activities may have acted as aggravating factors for the impacts of the pandemic on indigenous populations before to the start of vaccinations. Recently, Laudares and Gagliardi [54] found a clear connection between deforestation and the spread of COVID-19 among indigenous peoples in Brazil, suggesting that the main mechanisms through which deforestation intensifies human contact between indigenous and infected people are illegal mining and conflicts. Thus, this study constitutes an attempt to correlate the processes of environmental disturbance, and the mechanisms of population accessibility with the incidence of cases and deaths of indigenous people by COVID-19 in DSEIs in the BLA. Although we know that most countries do not officially recognize their indigenous groups and have inaccurate or unpublished statistical data about them, generating sparse systematic information on health, morbidity, and mortality [55], we assessed the effects based on data about COVID-19 cases and deaths among the indigenous population that was made available by a government agency. In the future, data made available by other sources, such as news outlets and research institutions, may clarify the relationships between the factors. Moreover, the poor application of existing environmental laws and the current policy of deconstruction of scientific institutions that investigate the acceleration of deforestation and the impact of COVID-19 on indigenous populations in Brazil constitute challenges to efforts that limit the environmental impacts of development activities and mortality by COVID-19 in the Brazilian Amazon. The current governance, based on the denial of isolation measures, the allocation of public resources for the production and distribution of drugs proven to be ineffective, and delays in vaccination, has driven the rise in the number of cases and deaths among indigenous populations in Brazil.

## Supporting information

S1 Data Dataset including information from DSEIs, number of inhabitants of cities associated with DSEIs and airports located in the area of each DSEI. (XLSX)

## References

[1] Silva SM. Management of Indigenous Health in Brazil: the Special Indigenous Sanitary Districts/DSEI’S and the Eastern District of Roraima. XVI SEMEAD Seminars in Administration. 2013; 16.

[2] Gracey M, King M. Indigenous health part 1: determinants and disease patterns. Lancet. 2009; 374: 65–75. doi: 10.1016/S0140-6736(09)60914-4 . 19577695

[3] Foschiera AA, da Silva JS. The spatialization of COVID-19 in special indigenous sanitary districts in the Legal Amazon. Geographic Atelier. 2020; 14:6–34.

[4] Fiocruz. Oswaldo Cruz Foundation. Risk of the spread of COVID-19 in indigenous populations: preliminary considerations on geographic and sociodemographic vulnerability. Rio de Janeiro: Fiocruz/ENSP/PROCC; FGV, 2020. 36 p. 4. Report, 18 April 2020.

[5] Silva Junior CHL, Person ACM, Carvalho NS, Reis JBC, Anderson LO, Aragão LEOC. The Brazilian Amazon deforestation rate in 2020 is the greatest of the decade. Nat Eco Evol. 2021; 5:144–145. doi: 10.1038/s41559-020-01368-x . 33349655

[6] INPE. National Institute for Space Research (INPE). Monitoring of the Brazilian Amazon deforestation by satellite. 2020. Available online: http://www.obt.inpe.br/OBT/assuntos/programas/amazonia/prodes (accessed on 14 April 2021).

[7] Mataveli GAV, Chaves MED, Brunsell NA, Aragão LEOC. The emergence of a new deforestation hotspot in Amazonia. Perspect Ecological Conservation 2021; 19:33–36. 10.1016/j.pecon.2021.01.002.

[8] Barlow J, Berenguer E, Carmenta R, France F. Clarifying Amazonia’s burning crisis. Glob Chang Biol. 2019; 26: 319–321. doi: 10.1111/gcb.14872 31729092

[9] Cardoso AM, Coimbra CEA, Werneck GL. Risk factors for hospital admission due to acute lower respiratory tract infection in Guarani indigenous children in southern Brazil: a population-based case-control study. Trop Med Int Health. 2013; 18:596–607. doi: 10.1111/tmi.12081 . 23489343

[10] Aragão LEOC, Silva Junior CHL, Anderson LO. Brazil’s challenge to restrain deforestation and fires in the Amazon during COVID-19 pandemic in 2020: environmental, social implications and their governance. National Institute for Space Research: São José dos Campos, Brazil, 2020. Available online: https://www.treeslab.org/uploads/4/6/5/4/465490/nt_desmatamento_fogo_e_covid-19_na_amazonia_-_circulacao.pdf (accessed on 1 October 2021).

[11] De Oliveira G, Chen JM, Stark SC, Berenguer E, Moutinho P, Artaxo P, et al. Smoke pollution’s impacts in Amazonia. Science. 2020; 369:634–635. doi: 10.1126/science.abd5942 . 32764060

[12] Yongjian Z, Jingu X, Fengming H, Liqing C. Association between short-term exposure to air pollution and COVID-19 infection: Evidence from China. Sci Total Environ. 2020; 727:138704. doi: 10.1016/j.scitotenv.2020.138704 . 32315904 PMC7159846

[13] Fattorini D, Regoli F. Role of the chronic air pollution levels in the Covid-19 outbreak risk in Italy. Environ Pollut. 2020; 264:114732. doi: 10.1016/j.envpol.2020.114732 . 32387671 PMC7198142

[14] Coelho MTP, Rodrigues JFM, Medina AM, Scalco P, Terribile LC, Vilela B, et al. Global expansion of COVID-19 pandemic is driven by population size and airport connections. PeerJ. 2020; 8:e9708. 10.7717/peerj.9708.

[15] Fellows M, Paye V, Alencar A, Nicácio M, Castro I, Coelho ME, et al. Under-reporting of COVID-19 cases among indigenous peoples in Brazil: a new expression of old inequalities. Front Psychiatry, 2021; 12:638359. doi: 10.3389/fpsyt.2021.638359 33912084 PMC8071995

[16] Kirby KR, Laurance WF, Albernaz AK, Schroth G, Fearnside PM, Bergen S, et al. The future of deforestation in the Brazilian Amazon. Futures. 2006; 38:432–453. 10.1016/j.futures.2005.07.011.

[17] Laurance WF, Albernaz AKM, Schroth G, Fearnside PM, Bergen S, Venticinque EM, et al. Predictors of deforestation in the Brazilian Amazon. J Biogeogr. 2002; 29:737–748. 10.1046/j.1365-2699.2002.00721.x.

[18] ESRI. ArcGIS 10.5. Environmental Systems Research Institute. Redlands, 2017 CA.

[19] Wei T, Simko V, Levy M, Xie Y, Jin Y, Zemla J, et al. Package ’corrplot’. 2021; https://cran.r-project.org/web/packages/corrplot/corrplot.pdf.

[20] R Core Team. A Language and Environment for Statistical Computing. R Foundation for Statistical Computing, Vienna, Austria. 2021. Available online: https://www.R.project.org/. (accessed on 4 July 2021).

[21] Fox J, Weisberg S, Price B, Adler D, Bates D, Baud-Bovy G, et al. R-Core. Package Car. 2019; https://cran.r-project.org/web/packages/car/index.html.

[22] Hadfield JD. MCMC methods for multi-response generalized linear mixed models: The MCMCglmm R package. J Stat Software 2010; 33:1–22. 10.18637/jss.v033.i02.

[23] Burnham KP, Anderson DR. Model selection and multimodel inference, 2nd ed.; Springer: New York, NY, USA, 2002.

[24] Zuur A, Ieno EN, Walker N, Saveliev AA, Smith GM. Mixed Effects Models and Extensions in Ecology with R; Springer Science & Business Media: Berlin/Heidelberg, Germany, 2009.

[25] Rodrigues D, Mendonça S. Old and new threats in Xingu. In: Ricardo F, Ricardo B, organizers. Indigenous peoples in Brazil 2011/2016. São Paulo: Instituto Socioambiental; 2017. p. 585–90.

[26] Fontão MAB, Pereira EL. Mais Médicos project in indigenous health: reflections based on an opinion poll. Interface (Botucatu). 2017; 21 Suppl 1:1169–80.

[27] Lopes P, Lopes A. The importance of Pap smear testing in primary health care units. InterSaúde Magazine. 2020; 1:129–140.

[28] Veiga ML, Sobrinho Junior AL, Barreto EL, Born KCCG, dos Reis LK, Brilhante RV, et al. Prevalence of breastfeeding in the municipality of Belém in three basic health units. Brazil J Health Rev. 2020; 3:9864–9874. 10.34119/bjhrv3n4-217.

[29] Monteiro MGPB, Rodrigues FFP, Pereira IC, Souza MVS, Miranda RP. The importance of the yellow September action in basic health units. Rev APS. 2020; 23:180–181.

[30] Oliveira MIC, Camacho LAB. Impact of basic health units on the duration of exclusive breastfeeding. Rev Bras Epidemiol. 2002; 5:41–51. 10.1590/S1415-790X2002000100006.

[31] Pine VFS, Coutinho ESF. Variables associated with breast cancer in clients of primary healthcare units. Public Health Notebooks. 2007; 23:1061–1069. 10.1590/s0102-311x2007000500008. 17486229

[32] Juliano Y, Compri PC, Almeida LR, Freire PV, Moreira FT, Vieira FHS, et al. Second stage of the national multivaccination campaign in the city of São Paulo, 2005: profile of coverage at different Health Centers. Rev Paul Pediatr. 2008; 26:14–9.

[33] Mendonça SBM, Rodrigues D, Pereira PPG. Indigenous health care model: the case of DSEI Xingu. Public Health Cad. 2019; 35:e00008119. doi: 10.1590/0102-311X00008119 31433028

[34] DSEI Xingu. DSEI Xingu Information Sector, Xingu Project—EPM/Unifesp. Number of deaths in children under one year of age per neonatal and post-neonatal period, DSEI Xingu 2011 to 2016. São Paulo: Federal University of São Paulo; 2018.

[35] Codeço CT, Villela D, Coelho F, Bastos LS, Carvalho LM, Gomes MFC, et al. Risco de espalhamento da COVID-19 em populações indígenas: considerações preliminares sobre vulnerabilidade geográfica e sociodemográfica. Rio de Janeiro, RJ (2020). Available online at: https://portal.fiocruz.br/documento/4o-relatorio-sobre-risco-de-espalhamento-da-covid-19-em-populacoes-indigenas (accessed December 6, 2021).

[36] BenYishay A, Heuser S, Runfola D, Trichler R. Indigenous land rights and deforestation: Evidence from the Brazilian Amazon. J Environ Economic Manager. 2017; 86:29–47. 10.1016/j.jeem.2017.07.008.

[37] Paiva PFPR, Ruivo MLP, da Silva Júnior OM, Maciel MNM, Braga TGM, de Andrade MMN, et al. Deforestation in protect areas in the Amazon: a threat to biodiversity. Biodivers Conservation 2019; 29:19–38. 10.1007/s10531-019-01867-9.

[38] Nolte C, Agrawal A, Silvius KM, Soares-Filho BS. Governance regime and location influence avoided deforestation success of protected areas in the Brazilian Amazon. Proc Natl Acad Sci. 2013; 110:4956–4961. doi: 10.1073/pnas.1214786110 23479648 PMC3612687

[39] Jusys T. Changing patterns in deforestation avoidance by different protection types in the Brazilian Amazon. PLoS One. 2018; 13:e0195900. doi: 10.1371/journal.pone.0195900 . 29689071 PMC5918171

[40] Amado LHE. Authoritarianism and the indigenous resistance in Brazil. Rev. Eletron. Comun. Inf. Inov. Saúde. 2019; 13:702–706. 10.29397/reciis.v13i4.1939.

[41] De Oliveira G, Chen JM, Mataveli GAV, Chaves MED, Seixas HT, Cardozo FS, et al. Rapid recent deforestation incursion into a vulnerable indigenous land in the Brazilian Amazon and fire-driven emissions of fine particulate aerosol pollutants. Forests. 2020; 11:829. 10.3390/f11080829.

[42] INPE. National Institute for Space Research (INPE). Portal TerraBrasilis, 2020. http://terrabrasilis.dpi.inpe.br.

[43] Ferrante L, Fearnside PM. Brazil’s new president and ’ruralists’ threaten Amazonia’s environment, traditional peoples and the global climate. Environmental Conservation 2019; 46: 261–263.

[44] Friend I. When will the Amazon hit a tipping point? Nature. 2020; 578:505–507. doi: 10.1038/d41586-020-00508-4 . 32099130

[45] Fiocruz. Oswaldo Cruz Foundation. COVID-19 and fires in the Legal Amazon and Pantanal: cumulative aspects and vulnerabilities. Rio de Janeiro: Fiocruz/ENSP/PROCC; FGV, 2021. Technical note, p. 17.

[46] Reporter Brasil. 2020. https://reporterbrasil.org.br/2020/11/integra-da-resposta-da-funai-sobre-focos-de-incendio-em-terras-indigenas/.

[47] By Miguel Buckley R, Díaz-Menéndez M. Go to gate: COVID-19 imported cases in Madrid and the potential role of airport transmissions. Trans R Soc Trop Med Hyg. 2021; 115:731–732. doi: 10.1093/trstmh/traa198 . 33444435 PMC7928668

[48] Laurance WF, Albernaz AKM, Costa CD. Is deforestation accelerating in the Brazilian Amazon? Environmental Conservation 2001; 28:305–311. 10.1017/s0376892901000339.

[49] Jusys T. Fundamental causes and spatial heterogeneity of deforestation in Legal Amazon. Appl Geogr. 2016; 75:188–199. 10.1016/j.apgeog.2016.08.015.

[50] Douglas RA. Low-volume road engineering: Design, construction, and maintenance. Boca Raton: Taylor & Francis Group. 2017. ISBN 9781138748156.

[51] Shirvani Z, Abdi O, Buchroithner MF. A new analysis approach for long‐term variations of forest loss, fragmentation and degradation from road‐network expansion using landsat time‐series resulting and OBIA. Land Degrad Dev. 2020; 1–20. 10.1002/ldr.3530.

[52] Chomitz KM, Gray DA. Roads, land use, and deforestation: A spatial model applied to Belize. World Bank Rev. 1996; 10:487–512. 10.1093/wber/10.3.487.

[53] Trombulak SC, Frissell CA. Review of ecological effects of roads on terrestrial and aquatic communities. Conservation Biol. 2000; 14:18–30. 10.1046/j.1523-1739.2000.99084.x.

[54] Laudares H, Gagliardi PH. Is deforestation spreading COVID-19 to the indigenous peoples? Covid Econ. Vetted. Real Time Pap. 2020; 53:33–71 (https://ieps.org.br/wp-content/uploads/2020/11/IEPS_WP8.pdf).

[55] Stephens C, Nettleton C, Porter J, Willis R, Clark S. Indigenous peoples’ health—why are they behind everyone, everywhere? Lancet. 2005; 366:10–13. doi: 10.1016/S0140-6736(05)66801-8 15993213

